# Health Policy Training: A Review of the Literature

**DOI:** 10.3390/ijerph13010020

**Published:** 2015-12-23

**Authors:** Harry J. Heiman, L. Lerissa Smith, Marissa McKool, Denise N. Mitchell, Carey Roth Bayer

**Affiliations:** 1 Satcher Health Leadership Institute, Morehouse School of Medicine, 720 Westview Drive, Atlanta, GA 30310, USA; llsmith@msm.edu; 2 Hubert Department of Global Health, Rollins School of Public Health, Emory University, GA 30322, USA; mmckool@emory.edu; 3 Health Policy Research and Innovation Program, American Institutes for Research, 1000 Thomas 19 Jefferson Street NW, Washington, DC 20007, USA; dmitchell@air.org; 4 Departments of Community Health and Preventive Medicine/Medical Education, Satcher Health Leadership Institute, Morehouse School of Medicine, 720 Westview Drive, Atlanta, GA 30310, USA; cbayer@msm.edu

**Keywords:** health policy, health policy training, training, education, curriculum, health professionals, health disparities, health equity, health in all policies, public health

## Abstract

The context within which health care and public health systems operate is framed by health policies. There is growing consensus about the need for increased health policy leadership and a health professional workforce prepared to assume these leadership roles. At the same time, there is strong evidence supporting the need for a broader policy lens and the need to intentionally target health disparities. We reviewed the published literature between 1983 and 2013 regarding health policy training. From 5124 articles identified, 33 met inclusion criteria. Articles varied across common themes including target audience, goal(s), health policy definition, and core curricular content. The majority of articles were directed to medical or nursing audiences. Most articles framed health policy as health care policy and only a small number adopted a broader health in all policies definition. Few articles specifically addressed vulnerable populations or health disparities. The need for more rigorous research and evaluation to inform health policy training is compelling. Providing health professionals with the knowledge and skills to engage and take leadership roles in health policy will require training programs to move beyond their limited health care-oriented health policy framework to adopt a broader health and health equity in all policies approach.

## 1. Introduction

Health policy provides the context and framework within which health care and public health systems operate. There is growing consensus regarding the need for increased health policy leadership and a health professional workforce prepared to assume these leadership roles. Health professional leaders and educators from medicine, nursing, public health, and other disciplines have advocated for the importance of health policy training to support engagement and leadership in public policy issues that impact their professions and the health of communities they serve [1,2,3,4,5,6,7,8]. The Institute of Medicine (IOM) highlighted the need for health policy training for health professionals in their 2003 report, *Who Will Keep the Public Healthy? Educating Public Health Professionals for the 21st Century*, stating “Should schools wish to be significant players in the future of public health and health care, dwelling on the science of public health without paying appropriate attention to both politics and policy will not be enough” [9]. This was reiterated by the Commission on Education of Health Professionals for the 21st Century, who noted in their 2010 report that “complementary requisite skills for (health leaders) should include key health system functions such as planning, policy, and management” [10]. 

Coincident with the increased focus on health policy, there has also been increased evidence and advocacy supporting the need for a broader policy lens and the need to intentionally target health disparities. In follow up to their earlier report, the IOM’s 2011 report, *For the Public’s Health: Revitalizing Law and Policy to Meet New Challenges*, emphasized the importance of leveraging public policy to improve population health and the need to adopt a health in all policies approach—an approach that not only looks at policies affecting the health care and public health systems, but also looks at the health effects of policies in non-health sectors [11]. The public health community strongly endorses health in all policies as a core strategy for advancing population health and health equity as reflected in key publications from the American Public Health Association [12] and the Association of Schools and Programs of Public Health’s (ASPPH) Expert Panel Report on *Population Health across All Professions* [13]. Reducing health disparities has been a core goal of the national Healthy People health promotion and disease prevention strategy since 1990 [14]. In *Healthy People 2020*, there is recognition that eliminating disparities and achieving health equity will require addressing not only health care-related disparities, but also structural and environmental factors, including social determinants of health [15]. This broad health policy and health equity lens is also reflected in the National Prevention Strategy [16] and National Partnership for Action to End Health Disparities [17]. We endorse this broad definition of health policy that includes governmental, organizational, and institutional policies, including policies in non-health sectors, that directly or indirectly impact health and health equity.

The goal of this review was to identify and analyze the published literature on health policy training, specifically in the context of the identified need for health policy training among health professionals and the increasing emphasis on broader approaches to policy, including population health, health equity, and health in all policies. The overarching hypothesis was that there was not a consensus in the published literature regarding the framework, scope, or core elements of health policy training. Based on this hypothesis and the evolving health policy framework, the following research questions were used to guide this review:

1. What literature exists regarding the development or implementation of formal health policy training and training programs?
2. How is “health policy” framed and defined, relative to the increasing focus on health in all policies?
3. What are the core components of health policy training?
4. What evaluation data and research exist to inform heath policy training?
5. What barriers exist to implementation of health policy training in health professional training programs?

In 1983, the American Psychological Association’s Working Group on Health Policy Training provided one of the early calls for the importance of health policy training [1]. Using this as a benchmark, we reviewed the published literature from 1983–2013 to assess any existing consensus regarding health policy training, answer the framing research questions above, and help inform the development of a shared framework for meeting the needs of current and future health policy leaders.

## 2. Methods

**Figure 1:**
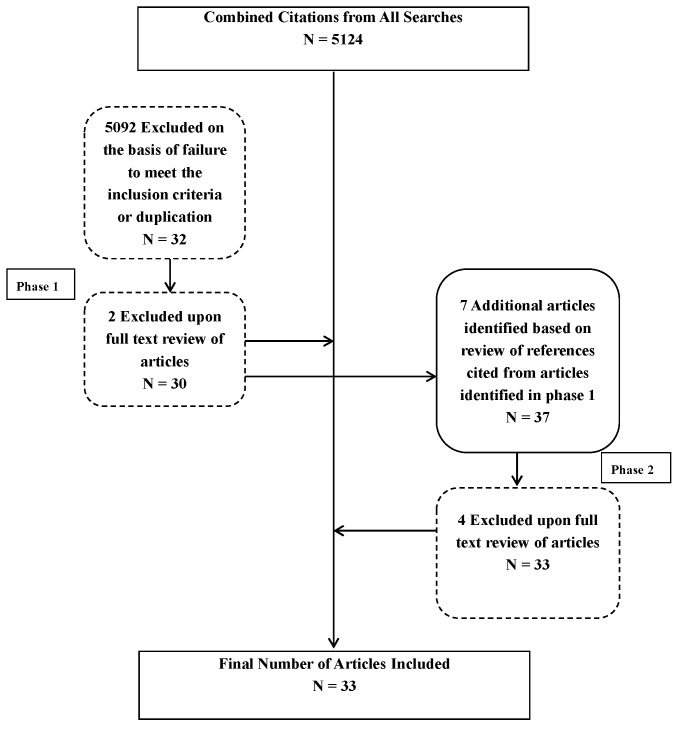
Search and article selection process and results. * Broken lines denote deductions; solid lines denote inclusion.

A systematic literature review was performed of the published literature between the years 1983 and 2013. Published literature included white papers, issue briefs, and publications in peer-reviewed journals. The literature search was performed using a multipronged approach. The first phase was conducted using a combination of key words including: health policy; training; education; curriculum; leadership; and controlled vocabulary terms in the following search engines or databases: PubMed, EBSCOHost, JSTOR, ERIC, DOAJ, and Google Scholar. Our search included literature from various disciplines including psychology, sociology, medicine, nursing, education, public health, and public policy. As reflected in Figure 1, a total of 5124 articles were identified during the first phase. Articles were selected for inclusion if they were written in English, published between 1983 and 2013, and had health policy training as a significant focus. Based on title and abstract review, application of the inclusion criteria, and deduplication of results, 32 articles were downloaded for full text review. During full text review, 2 additional articles were excluded based on lack of relevance to health policy training. The second phase of article identification involved a review of references cited in the selected articles; an additional 7 articles were reviewed from these references, 3 of which were selected for inclusion. A total of 33 articles were included in the final literature review. 

Content analyses were performed to identify and group information categories relevant to the hypothesis and research questions outlined above. An abstraction template was then developed based on these categories.

## 3. Results

After synthesizing and conducting content analyses of the research literature, six main categories emerged: target audience, goal(s) of the article, definition of health policy, core components of health policy training, evaluation methodology and results, and barriers to health policy training.

### 3.1. Target Audience

The majority of articles identified from the literature search were targeted toward medical audiences—medical students, residents, or medical school faculty [4,8,18,19,20,21,22,23,24,25,26,27,28] (39%, n = 13) or nursing audiences—nursing students or nursing school faculty [5,6,29,30,31,32,33,34,35,36,37,38] (36%, n = 12). Fewer articles were directed to public health professionals and students [7,39,40] (9%, n = 3), health administration and management faculty and students [41,42] (6%, n = 2), psychology faculty and students [1] (3%, n = 1), public policy and public administration faculty and students [43] (3%, n = 1), and a combination of business, public policy, and health administration faculty and students [44] (3%, n = 1). 

### 3.2. Goal(s) of the Articles

Four major themes or goals emerged in the analysis, with some articles addressing multiple areas: (1) description of proposed health policy training elements or curriculum; (2) pilot of a health policy training program; (3) advocacy for increased health policy training; and/or (4) survey of health policy training in a particular health professional curriculum. Most of the articles identified with more than one goal included advocacy for health policy training in addition to a different primary goal. These topics are discussed in greater detail below. 

#### 3.2.1. Description of Proposed Health Policy Training Elements or Curriculum

Six articles (18%) described a proposed framework or curriculum for health policy training directed toward a defined audience of learners [1,8,26,39,42,44]. These included training for undergraduate, graduate, or post-graduate learners across a range of disciplines. Authors provided recommendations regarding both the content and teaching methods they felt were most important and most effective. These included a range of proposed didactics as well as recommendations regarding the use of case studies and experiential learning in policy settings. 

#### 3.2.2. Pilot of a Health Policy Training Program 

Sixteen articles (48%) proposed and piloted a health policy training program for a defined set of learners, primarily a specific group of heath professional trainees, either in a particular venue, such as a hospital or community-based setting, or as a health policy component added or integrated into a training program. These programs varied in intensity and time commitment, ranging from a single “Health Care Reform Day” for medical students [4] to a three week intensive [20] or two year-long integrated curriculum [23] for medical residents. Similarly, nursing programs ranged from policy-oriented service learning programs over 2–3 semesters [31,32,36] to multidisciplinary programs in health policy for nursing masters and doctoral level students [34]. As discussed in the Evaluation section below, 75% of these (12 of 16) included evaluation results, though they were generally quite limited in scope.

#### 3.2.3. Advocacy for Increased Health Policy Training

Thirteen articles (39%) specifically included advocacy for increased health policy training. Of these, advocating for the importance of health policy training was the primary goal in four articles, three targeted to medical audiences [18,19,21] and one targeted to nursing audiences [5]. The other nine articles included advocacy for the importance of health policy training to support or complement a curricular description, pilot, or survey results directed to medical, nursing, public health, and psychology students [1,4,6,7,8,26,29,37,38]. 

#### 3.2.4. Survey of Health Policy Training

Seven articles (21%) presented survey results assessing health policy training. Survey methodologies ranged from course catalogue and curricular reviews [7,41,43] to surveys of deans [25] and graduate program directors [6]. Surveys covered a range of program and learner disciplines, including medicine, nursing, public health, public policy and public administration, and health services management.

### 3.3. Definition of Health Policy

All of the articles included in the review directly or indirectly provided a working definition of health policy for purposes of framing health policy training. The vast majority (79%, n = 26) defined health policy as health care policy, specifically framing it around content and policies related to the health care delivery system. Of these 26 articles, three also explicitly included public health policy [1,20,22] (one of which included social determinants of health) [20], one included policy and administration [43], and one included health services research [32]. The predominate focus, however, remained on policies that impact the health care delivery system. Two articles (6%) directed to public health audiences defined health policy as public health policy [30,40]; two articles (6%), one directed to medical trainees and the other to health administration and management trainees, defined health policy as health administration and management [18,41]; and three articles (9%) adopted a broader health in all policies definition [21,36,37]. These included not only health care delivery and public health, but also the health impact of policies in non-health sectors. Of these, one was directed to medical students and advocated for an expanded view of health policy that includes political, social, and economic policies and the nonmedical determinants of health [21]. The other two were directed to nursing students and emphasized the importance of “health-enhancing public policies” that extend beyond the health sector [37] and “healthy public policy” that includes a focus on social justice and health disparities [36].

### 3.4. Core Components of Health Policy Training

Almost all of the articles (91%, n = 30) included a discussion or description of components of health policy training. In the articles presenting survey results, authors included summaries of curricular components. In all of the articles, this information was framed by the overall goal of the article and included process components, focused on teaching format and methods, and content components, presented as curricular topics or areas of core curricular content. 

#### 3.4.1. Process Components

Several common themes related to the format and methods of health policy training emerged. Those discussed in multiple articles are summarized in Table 1. While training often included didactics, a number of articles stressed the value of mixing lectures with other teaching formats, including case studies [25,28,42], seminars [28,34], site visits [20,30], group projects [28,30], and discussion sessions [28]. The value of including policy experts and politicians as lecturers and subject matter experts was also emphasized [34,35,37]. Some programs also stressed the importance of policy-related skill-building and included writing position papers, writing legislative bills, drafting mock testimonies for a hearing [35], and a culminating experience of a mock U.S. congressional hearing in which students testified as subject matter experts [40]. In an effort to integrate health policy training into clinical training, one program introduced policy-relevant questions into hospital ward rounds to identify potential barriers to patient and community health [22]. 

**Table 1:** Common curricular process components of health policy training.

Process Components	References
Experiential/service learning	[27,31,32,33,34,36,37,38]
Case studies	[25,28,42]
Inclusion of policy experts and politicians	[34,35,37]
Site visits	[20,30]
Group projects and discussion sessions	[28,30]
Seminars	[28,34]
Policy-related skill building	[35,40]

A number of articles emphasized the value and importance of experiential or service learning. Of the sixteen articles that described pilots of training programs, eight included placement in an organizational, agency, or community setting with a defined role or policy-relevant project [27,31,32,33,34,36,37,38]. Of these eight, seven were implemented in graduate nursing programs. Variably named, these experiences included a residential fellowship in a congressional office, agency, or advocacy organization in Washington, D.C., USA [27], a health policy residency, practicum, or internship in a health organization or agency [31,32,33,34,37], and group community-based service learning projects addressing policy-relevant clinical or community health issues [36,38]. A common characteristic of all of these programs was the importance of engaging students in real world settings that allowed them to grapple with policy issues relevant to population and community health and their careers as health professionals.

#### 3.4.2. Content Components

A broad range of content components was discussed in the articles. These were grouped thematically and repeated themes and subtopics summarized in Table 2. The most common core content components included health care costs and financing [6,24,25,26,28,29,33,35], current trends or topics in health policy [4,23,24,25,28,29,33,36], health care structure and organization [1,8,20,23,26,29,35], quality assessment and assurance [8,24,25,28,29,35], the policy process [6,26,34,37,39,40], population health dimensions of health policy [21,29,35,36,37], health policy for vulnerable populations [8,20,24,36], ethical and legal issues in health policy [1,24,40], policy communication and advocacy [34,37,40], policy and politics [6,8,20], and the physician role in health policy and health system reform [20,23]. Additional topics noted included the health workforce, medicine and technology, licensing and accreditation, institutional accountability, physician human resource planning, technical aspects of public health, global health, and quantitative and qualitative research.

**Table 2:** Common curricular content components of health policy training *.

Content Components	References
**Health care costs and financing**	[6,24,25,26,28,29,33,35]
Cost containment and resource allocation	[6,26,29,33,35]
Physician payment systems	[28]
Medicare and Medicaid	[25]
Physician reimbursement and insurance design	[24,25]
**Current trends or topics in health policy**	[4,23,24,25,28,29,33,36]
Health care reform	[4,25]
The Affordable Care Act and U.S. Supreme Court decision	[24]
The Nurse Practice Act	[36]
**Health care structure and organization**	[1,8,20,23,26,29,35]
Health care systems and principles	[8]
Introduction to Canada’s healthcare system	[23]
Organization of clinical and public health systems	[26]
**Quality assessment and assurance**	[8,24,25,28,29,35]
Quality improvement	[25]
Quality and safety	[8]
Health quality and health status	[24]
**Policy process**	[6,26,34,37,39,40]
Health policy overview	[6]
Health policy theory and health policy analysis	[39]
Policy-making intervention points	[40]
**Population health dimensions of health policy**	[21,29,35,36,37]
Impact of political, economic, and social policies on health	[21]
Community-level determinants of health	[36]
Community health assessments	[36]
Population health promotion	[36]
**Health policy for vulnerable populations**	[8,20,24,36]
Health disparities	[8,20]
Health care safety net	[8,20]
Social determinants impacting health	[20]
Civil rights and health care	[20]
Access to health insurance	[24]
Social justice and social activism	[36]
**Ethical and legal issues in health policy**	[1,24,40]
Laws related to lobbying and advocacy for non-profits	[40]
**Policy communication and advocacy**	[34,37,40]
Public health advocacy and media advocacy	[40]
Media advocacy	[37]
**Policy and politics**	[6,8,20]
The politics of health care	[20]
Health politics and law	[8]
Policy versus politics	[6]
**Physician role in health policy**	[20,23]
Physician role in health systems reform	[23]

Note: * Table includes common curricular content themes as well as examples of related topics that fall under the theme.

### 3.5. Evaluation

Fourteen articles (42%) included discussion about evaluation methods or results. Evaluation descriptions ranged from mention of integrated formative and summative evaluation, with no sharing of evaluation measures, metrics, or results [34] or that they were “achieving important initial success” [33], to reporting that, based on alumni surveys, most graduates were working in health policy or policy-related positions [31]. In general, evaluation information was quite limited, focused much more on course/program evaluations and student self-assessments of learning, attitude changes, and satisfaction, than on measuring the impact of policy training on policy involvement or learner career paths.

### 3.6. Barriers to Health Policy Training

Ten articles (30%) discussed barriers either anticipated or experienced in implementing health policy training. All of these reflected the challenges of integrating health policy into clinical training programs (medicine, nursing, and psychology). Common themes included the lack of perceived relevance, the lack of resources (or concerns regarding competition for finite resources), and lack of faculty expertise and interest [25], including concerns regarding the challenges of recruiting faculty with policy expertise and the lack of access to interdisciplinary faculty. Barriers also included scheduling and time constraints—specifically concerns that policy training would compete with and dilute and distract from core clinical training [31]. Patel and colleagues also expressed concerns about the lack of research to inform implementation of health policy curricula [8]. There were specific challenges discussed regarding experiential learning. These included identification of placement sites, site readiness, addressing problems during the practicum, engaging stakeholders, confidentiality and ethical approval issues, and inadequate time [32]. 

## 4. Discussion

Despite the increased interest and consensus regarding the importance of health policy training, literature published in the past 30 years on health policy training remains limited. This may reflect, in part, that health policy training frequently develops and evolves within and across educational settings without rigorous research, evaluation, or publication of methods. As a result, non-indexed sources like syllabi and course and program descriptions likely exist, but are not accessible through a literature search. Despite the limited published literature, however, there are important findings that help inform health policy training and frame future research.

The existing health policy training literature is primarily targeted to medical and nursing audiences. Given the number of health policy and management programs in schools of public health and the spectrum of health policy fellowship programs, some of which have been in place for over 40 years, the lack of a more robust literature, especially from public health training programs, is surprising and notable. Given ASPPH’s clear framework of essential content domains for training in population health and health in all policies [13], there is a compelling need for program evaluation and research to inform training implementation. 

Having a commonly agreed upon definition of health policy is critical to framing the core elements, training modalities, and goals of health policy training. In contrast to our broad definition and the health in all policies approach being embraced by the public health community, the majority of articles reviewed framed and defined health policy through the narrow lens of health care policy. While it was expected that definitions would reflect the disciplinary perspective of the authors, it is concerning that at a time when the need for a broader policy lens is recognized across health professional disciplines, the focus remains relatively narrow. Even in more recent publications and in fields such as public health, where one would expect a broader perspective, health policy was still defined in the context of the health care system. This likely reflects the disproportionate attention that health care and health services research have traditionally received in health policy training programs. Preparing health policy practitioners to move beyond current silos, not only within health care, but also between health care, public health and the spectrum of non-health sectors that impact population health will require both a multidisciplinary lens and a broader health in all policies definition and approach.

A number of common themes regarding core curricular content and teaching methods were identified as reflected above and in Table 2. Given the overarching focus on health care policy, it was not surprising that in addition to fundamentals regarding the policy process, core curricular components also included health care finance, organization, and quality. To ensure relevance, many programs also included current topics and trends, such as health care reform. A limited number of authors included specific content on population health and vulnerable populations. 

The lack of curricular content related to health disparities and health equity is noteworthy, especially given the importance of health equity to national public health planning and policies [15,17]. Content including underserved populations, health disparities, or health equity was found in only four articles (12%), none of which were directed to public health audiences [8,20,34,36]. Given the persistence of health disparities in both the U.S. and globally, relevant content, including the emerging literature on evidence-based policy approaches to reduce disparities and advance health equity should be included in core curricular content across health policy training programs [12,45]. 

The value of interactive, experiential learning was stressed by a number of articles. Creating opportunities for trainees to be exposed to content through discussion of case studies, interactions with health policy practitioners and leaders, and real world practical experiences in policy-relevant settings were felt to be valuable to the training process. Leveraging educational experiences that provide opportunities to engage in practical applications of their training were critical to ensuring the translation and relevance of health policy training.

The literature was extremely limited with regard to evaluation data to inform health policy training. Less than half of the articles reviewed mentioned evaluation and of those, the focus was primarily on short-term evaluations and self-reported assessments. The lack of a comprehensive framework, including delineated goals and learner objectives to evaluate training and learner outcomes, and the lack of longer-term outcome and impact data, limited our ability to identify best and evidence-based practices both within and across subsets of health policy learners.

Establishing a core training framework and evaluation matrix that guides training and evaluation across programs and disciplines is essential. Ensuring that evaluation moves beyond assessments of course content learning, learner self-assessment, and course/program satisfaction to assessment of policy-relevant longitudinal measurements of programmatic outcomes is critical. Defining the range of relevant health policy roles and the necessary knowledge, skills, and experiences is an important initial step. Evaluating how health policy training informs and impacts learners’ careers as they engage and intersect with health policy-relevant or related experiences is imperative. Without meaningful systematic evaluation, programs will continue to lack the ability to identify best practices and measure program impact. 

The identified barriers to health policy training reflect a longstanding culture, especially in clinical disciplines, that protects the status quo. Chief among these were the lack of apparent relevance, lack of health policy capacity among faculty, the perceived lack of resources and time, and concerns about the impact on other competing training needs and priorities. Like other system-level changes in health and health care that require change on the part of health professional educators, recognizing and responding to the need for health policy training will require reassessing and prioritizing the needs of students and future practitioners. 

Consistent with the recommendations of the Commission on Education of Health Professionals for the 21st Century [10], health policy training programs will need to reach across academic institutions and outside institutional walls to engage the necessary interdisciplinary and community- and agency-based partners and experts to support health policy training needs. While the lack of interdisciplinary faculty was an identified barrier to implementing health policy training, it was also a noted strength of many programs presented. The recognition of the value of engaging outside experts and policy practitioners as well as partnering to support policy-relevant community-based learning experiences reflects the consensus regarding the need to build both internal capacity and collaborative external partners. 

As with all literature reviews, this review has inherent limitations. As noted above, training programs, curricula, and syllabi developed within academic settings may not have been captured in a literature search. In addition, it is possible that topic areas related to population health, social determinants of health, and health disparities are covered in other training program curricula, but not captured under health policy. While both search terms and search engines were selected with the goal of capturing the relevant literature across a spectrum of disciplines, it is possible that additional relevant key words or search engines were inadvertently excluded. Given the lack of consensus regarding the scope and meaning of the term “health policy” and the potential subjectivity in applying inclusion criteria and core themes, relevant articles may have been excluded and or miscategorized. Given that our research team included professionals from a variety of disciplines, this limitation should have been minimized. While a survey of program directors or deans across health professional training programs might have captured a more complete picture of the health policy training landscape, this was beyond the scope of this literature review. Finally, the equal weighting of articles summarizing survey data and articles describing single programs with regard to identifying themes and trends may slightly skew results. Understanding these limitations, there remain a number of important conclusions that bear further discussion.

## 5. Conclusions

The U.S. continues to underperform with regard to health and health care outcomes relative to other developed countries. There are also persistent health disparities experienced not only by low income and racial and ethnic minority populations, but also rural populations, sexual minority populations, people living with disabilities, and others who experience social, economic, and environmental disadvantage. Policy change is a critical strategy to advancing both health and health equity. Health professional leaders and educators across disciplines have acknowledged the need for increased health policy training and leadership. Consistent with our hypothesis, there is no collective framework for designing or implementing health policy training. In addition, few programs have adopted the broader health in all policies lens or integrated content or approaches that directly address health disparities or health equity.

Based on our review of the literature from 1983 to 2013, there is a compelling need for more rigorous research and evaluation to inform health policy training. Building health policy capacity across learners will require educators to adopt a broad, interdisciplinary lens and move outside the walls of academia. In addition, training programs will need to move beyond their limited health care-oriented health policy framework to adopt a broader health and health equity in all policies lens. Health professionals are increasingly aware that health care, though essential, is a relatively weak determinant of health [46]. Framing and informing health policy training with a clear health policy definition and lens that acknowledges the importance of non-health sectors in driving population health is essential to meeting the training needs of current and future health professionals and leaders. It is also critical to ensuring that they have the necessary knowledge, skills, and experiences to advance the health and health equity of our nation. 
